# Long-term yield of pancreatic cancer surveillance in high-risk individuals

**DOI:** 10.1136/gutjnl-2020-323611

**Published:** 2021-04-05

**Authors:** Kasper A Overbeek, Iris J M Levink, Brechtje D M Koopmann, Femme Harinck, Ingrid C A W Konings, Margreet G E M Ausems, Anja Wagner, Paul Fockens, Casper H van Eijck, Bas Groot Koerkamp, Olivier R C Busch, Marc G Besselink, Barbara A J Bastiaansen, Lydi M J W van Driel, Nicole S Erler, Frank P Vleggaar, Jan-Werner Poley, Djuna L Cahen, Jeanin E van Hooft, Marco J Bruno, the Dutch Familial Pancreatic Cancer Surveillance Study Group, M J Bruno

**Affiliations:** 1 Department of Gastroenterology & Hepatology, Erasmus MC Cancer Institute, University Medical Center Rotterdam, Rotterdam, The Netherlands; 2 Division Laboratories, Pharmacy and Biomedical Genetics, Department of Genetics, University Medical Center Utrecht, Utrecht, The Netherlands; 3 Department of Clinical Genetics, Erasmus MC Cancer Institute, University Medical Center Rotterdam, Rotterdam, The Netherlands; 4 Department of Gastroenterology & Hepatology, Amsterdam Gastroenterology Endocrinology Metabolism, Amsterdam UMC, University of Amsterdam, Amsterdam, The Netherlands; 5 Department of Surgery, Erasmus MC Cancer Institute, University Medical Center Rotterdam, Rotterdam, The Netherlands; 6 Department of Surgery, Cancer Center Amsterdam, Amsterdam UMC, University of Amsterdam, Amsterdam, The Netherlands; 7 Department of Biostatistics, Erasmus MC Cancer Institute, University Medical Center Rotterdam, Rotterdam, The Netherlands; 8 Department of Gastroenterology & Hepatology, University Medical Center Utrecht, Utrecht, The Netherlands

**Keywords:** pancreatic cancer, surveillance, family cancer, endoscopic ultrasonography, magnetic resonance imaging

## Abstract

**Objective:**

We aimed to determine the long-term yield of pancreatic cancer surveillance in hereditary predisposed high-risk individuals.

**Design:**

From 2006 to 2019, we prospectively enrolled asymptomatic individuals with an estimated 10% or greater lifetime risk of pancreatic ductal adenocarcinoma (PDAC) after obligatory evaluation by a clinical geneticist and genetic testing, and subjected them to annual surveillance with both endoscopic ultrasonography (EUS) and MRI/cholangiopancreatography (MRI/MRCP) at each visit.

**Results:**

366 individuals (201 mutation-negative familial pancreatic cancer (FPC) kindreds and 165 PDAC susceptibility gene mutation carriers; mean age 54 years, SD 9.9) were followed for 63 months on average (SD 43.2). Ten individuals developed PDAC, of which four presented with a symptomatic interval carcinoma and six underwent resection. The cumulative PDAC incidence was 9.3% in the mutation carriers and 0% in the FPC kindreds (p<0.001). Median PDAC survival was 18 months (range 1–32). Surgery was performed in 17 individuals (4.6%), whose pathology revealed 6 PDACs (3 T1N0M0), 7 low-grade precursor lesions, 2 neuroendocrine tumours <2 cm, 1 autoimmune pancreatitis and in 1 individual no abnormality. There was no surgery-related mortality. EUS detected more solid lesions than MRI/MRCP (100% vs 22%, p<0.001), but less cystic lesions (42% vs 83%, p<0.001).

**Conclusion:**

The diagnostic yield of PDAC was substantial in established high-risk mutation carriers, but non-existent in the mutation-negative proven FPC kindreds. Nevertheless, timely identification of resectable lesions proved challenging despite the concurrent use of two imaging modalities, with EUS outperforming MRI/MRCP. Overall, surveillance by imaging yields suboptimal results with a clear need for more sensitive diagnostic markers, including biomarkers.

**Significance of this study**

**What is already known on this subject?**

- The prognosis of pancreatic ductal adenocarcinoma (PDAC) is dismal and greatly depends on the stage at detection.
- Survival may be improved by early detection through imaging-based surveillance of selected high-risk individuals who carry genetic mutations or in familial pancreatic cancer (FPC) kindreds.
- It is unknown if outcomes differ between the distinct risk groups and which imaging modality performs best.

**What are the new findings?**

- This study demonstrates a substantial cumulative incidence of PDAC (9.3%) in PDAC susceptibility gene mutation carriers but a negligible (0%) incidence in mutation-negative FPC kindreds.
- Of 10 patients who developed PDAC, 6 had resectable cancers, of which 3 were diagnosed at an early stage (T1N0M0). The other four presented as symptomatic metastasised interval carcinomas.
- Overall, the median survival of patients with PDAC was 18 months (range 1–32).
- Endoscopic ultrasonography (EUS) detected more solid lesions than MRI/magnetic resonance cholangiopancreatography (MRI/MRCP), and performing MRI/MRCP did not add relevant diagnostic value.

**Significance of this study**

**How might it impact on clinical practice in the foreseeable future?**

- Questions are raised about the eligibility of mutation-negative FPC kindreds for PDAC surveillance.
- For PDAC susceptibility gene mutation carriers, surveillance potentially leads to a diagnosis at an earlier stage, although a survival benefit cannot be demonstrated.
- Even with EUS outperforming MRI/MRCP, imaging-based surveillance did not convincingly demonstrate early recognition of relevant lesions leading to ample opportunity for timely treatment. Other diagnostic modalities including biomarkers should be explored to improve the outcome of surveillance in individuals at high risk of developing PDAC.

## Introduction

Pancreatic ductal adenocarcinoma (PDAC) is a deadly disease for which surgery combined with systemic chemotherapy provides the only chance for long-term survival. Unfortunately, the diagnosis is often established at an unresectable stage.^1^ Screening may decrease mortality through early detection,^2 3^ but in the absence of reliable biomarkers, it currently solely depends on imaging.^4^ This, in addition to a low incidence, renders population-wide PDAC screening unfeasible.^5^ Thus, at this time, the potential benefits of screening do not outweigh the potential harms, and screening for PDAC in asymptomatic adults in the general population is not recommended.^6^

Surveillance is therefore restricted to research programmes involving selected individuals with an increased familial PDAC risk (high-risk individuals), defined by the presence of a strong family history (familial pancreatic cancer (FPC) kindreds) or a proven germline pathogenic variant of a known PDAC susceptibility gene (henceforth referred to as mutation carriers). Surveillance may be beneficial in these high-risk individuals, but they are a heterogeneous target group, with a wide range of lifetime PDAC risks. Mutation carriers have an estimated relative risk from 2.7 to 132, depending on the gene involved.^7^ For FPC kindreds who are not genetically tested, the risk is estimated to be much lower, ranging from twofold in case of a single affected relative,^8–10^ to fourfold with two affected relatives,^8 11^ and up to 32-fold in case of three affected first-degree relatives.^11^ The risk may increase further with a younger age of PDAC-onset in the family.^12^ As a result, the yield and possible benefits of surveillance may differ between risk categories.

To date, large and long-term studies in high-risk individuals are scarce, and the knowledge of the effect of surveillance on PDAC-related mortality and morbidity therefore remains limited.^5^ In addition, while experts agree that either endoscopic ultrasonography (EUS) or MRI/magnetic resonance cholangiopancreatography (MRI/MRCP) should be used for PDAC surveillance,^4^ few studies have compared both modalities.^13–17^

Therefore, this study had three objectives: (1) to determine the long-term yield of pancreatic cancer surveillance in a large cohort of high-risk individuals; (2) to identify predictors for developing PDAC including carriership of a gene mutation and (3) to compare the sensitivity of EUS and MRI/MRCP for the detection of pancreatic abnormalities.

## Methods

### Study design

This study is part of the ongoing Dutch Familial Pancreatic Cancer surveillance study, a multicentre prospective cohort study performed in three university hospitals in the Netherlands. All participants gave written informed consent prior to enrolment and this manuscript was written according the Strengthening the Reporting of Observational Studies in Epidemiology (STROBE) guideline.^18^ Patients or the public were not involved in the design, or conduct, or reporting, or dissemination plans of this study.

### Participants

We included asymptomatic individuals with an estimated 10% or greater lifetime risk of PDAC, encompassing carriers of a germline mutation in a known PDAC susceptibility gene (*CDKN2A, LKB1/STK11, BRCA2, BRCA1, PALB2, TP53, MLH1, MSH2, MSH6, ATM*) and individuals without a known gene mutation, but with a family history of PDAC in at least two blood relatives, defined as FPC kindreds. Surveillance started at age 45 until 2013 and age 50 thereafter, or 10 years younger than the age of the youngest relative diagnosed with PDAC. Surveillance ended at age 75. The detailed inclusion and age criteria are listed in the online supplemental file 1.

10.1136/gutjnl-2020-323611.supp1 Supplementary data

Individuals were recruited by all clinical genetics departments in the Netherlands to which families were referred, or which they visited at their own initiative. They underwent detailed evaluation of the family history, verification of cancer diagnoses by review of medical records and genetic testing. Genetic testing was performed on a PDAC index case whenever possible and otherwise in a healthy first-degree relative. If a mutation in a PDAC susceptibility gene was found in a family member affected by PDAC, only family members who tested positive were enrolled.

### Surveillance protocol

Participants underwent annual surveillance with both EUS and MRI/MRCP at each visit. MRI/MRCP was performed with intravenous administration of gadobutrol, and images were reviewed by radiologists specialised in abdominal imaging. EUS was performed under conscious sedation with midazolam/fentanyl or deep sedation with propofol by a small group of highly experienced endosonographists (>1000 procedures; PF, BAJB, LMJWvD, FPV, JWP, JEvH and MJB). EUS was performed before the radiologist reported on the MRI/MRCP, and radiologists were not aware of the EUS findings when reviewing the MRI/MRCP. Radiologists and endosonographists were not blinded for the results of previously performed EUS and MRI/MRCP. Additional diagnostic modalities including CT, SonoVue contrast during EUS and fine-needle aspiration (FNA) were used on indication only.

Clinical management was decided on by a multidisciplinary expert panel consisting of gastroenterologists, radiologists, surgeons and pathologists and was as follows: (1) annual surveillance in case of no abnormalities, minor signs of chronic pancreatitis, or cystic lesions without worrisome features; (2) surveillance after 3 or 6 months when a concerning lesion not warranting immediate surgery was detected, as judged by the expert panel. These included indeterminate lesions (hypoechoic or hypointense lesions of unknown significance), cystic lesions with a worrisome feature but no high-risk stigmata^19^ and dilated main pancreatic ducts without a visible mass, except for the suspicion of a main duct intraductal papillary mucinous neoplasm (IPMN). If a lesion was no longer considered suspect for malignancy, surveillance was resumed at 12-month intervals; (3) surgical resection if the expert panel agreed on a high suspicion for malignancy, based on a solid lesion, positive cytology, main pancreatic duct dilation ≥10 mm and/or an abrupt calibre change or cystic lesions with high-risk stigmata or ≥2 worrisome features.^19^

### Study endpoints and statistical analysis

Study endpoints were: the cumulative incidence of PDAC after 5 and 10 years of follow-up, median survival, surgical resection rate, surgery-related mortality and the sensitivity of imaging modalities to detect pancreatic abnormalities. Differences in baseline characteristics were assessed using the independent samples T test, the Mann-Whitney U test and the χ² test. The cumulative PDAC incidence and the median survival were assessed using the Kaplan-Meier method; differences in cumulative PDAC incidence between genetic risk categories were analysed with the log-rank test. To identify possible predictors for developing PDAC, we compared groups using the Mann-Whitney U test and the Fisher’s exact test. To determine the sensitivity of EUS and MRI/MRCP in detecting pancreatic abnormalities, we used the presence of an abnormality on either EUS, MRI/MRCP, or CT as reference standard. We compared the sensitivities at the visits at which the abnormalities were first diagnosed using McNemar’s test. P<0.05 (two-sided) was considered statistically significant for all analyses. Statistical analysis was performed using SPSS Statistics 22 (IBM, Armonk, New York, USA).

## Results

### Participants and follow-up

Since October 2006, 366 asymptomatic high-risk individuals from 214 families had undergone the baseline surveillance examination (table 1). A total of 165 (45%) were proven mutation carriers and 201 (55%) were classified as mutation-negative FPC kindreds: 99 (49%) based on a mutation-negative PDAC index case and 38 (19%) based on a mutation-negative healthy FDR because the index case could not be tested. For 55 individuals (27%), information on who had been tested could not be retrieved and in 9 cases (4% of the FPC kindreds, 2.5% of the total cohort) genetic testing was not performed. Of the total cohort, 157 (43%) were men and the mean age was 54 (SD 9.9) years at enrolment and 59 (SD 9.6) years at the last visit before analysis.

**Table 1:** Baseline characteristics, stratified by genetic risk category

	Mutation carriers (n=165)	FPC kindreds (n=201)	P value
Age, mean (SD), years	52 (9.6)	56 (9.8)	<0.001
Male sex, n (%)	73 (44)	84 (42)	0.64
BMI, median (IQR)	25 (4.5)	25 (5)	0.46
Gene mutation, n (%)			–
*CDKN2A* p16	96 (58)	–	–
*BRCA2* + ≥2 blood relatives with PDAC	45 (27)	–	–
*BRCA2* + ≥1 FDR with PDAC	25	–	–
*BRCA2* + ≥1 SDR with PDAC but no FDR with PDAC	9	–	–
*BRCA2* without FDR or SDR with PDAC	11	–	–
*BRCA1* + ≥2 blood relatives with PDAC	7 (4)	–	–
*BRCA1* + ≥1 FDR with PDAC	6	–	–
*BRCA1* + ≥1 SDR with PDAC but no FDR with PDAC	1	–	–
*PALB2* + 3 blood relatives with PDAC	2 (1)	–	–
*STK11/LKB1*	9 (6)	–	–
*TP53* + 2 blood relatives with PDAC	5 (3)	–	–
*TP53* + ≥1 FDR with PDAC	2	–	–
*TP53* + ≥1 SDR with PDAC but no FDR with PDAC	3	–	–
*ATM* + 3 FDR with PDAC	1 (1)	–	–
Total number of any degree blood relatives with PDAC, n (%)			<0.001
0	32 (19)	–	–
1	24 (15)	–	–
2	49 (30)	104 (52)	–
3 or more	56 (34)	96 (48)	–
Age youngest blood relative with PDAC, median (IQR), years	49 (16)	56 (15)	<0.001
Blood relative with PDAC <50 years of age, n (%)	65 (39)	58 (29)	<0.001
Personal history of non-pancreatic malignancy, n (%)	81 (49)	24 (12)	<0.001
Personal history of diabetes mellitus, n (%)	3 (2)	13 (7)	0.03
Personal history of acute pancreatitis, n (%)	0 (0)	7 (4)	0.02
Smoking, n (%)			0.32
Never	78 (47)	101 (50)	–
Former	68 (41)	67 (33)	–
Current	17 (10)	31 (15)	–
Alcohol use, n (%)			0.88
Never	39 (24)	42 (21)	–
Former	7 (4)	11 (6)	–
Current	116 (70)	145 (72)	–

By definition, FPC kindreds do not carry any of the known PDAC susceptibility gene mutations and have two or more blood relatives affected with PDAC. For the total number of blood relatives with PDAC, smoking and alcohol use, percentages may not add up to 100% because of missing data in up to 2% of participants.

FDR, first-degree blood relative; FPC, familial pancreatic cancer; PDAC, pancreatic ductal adenocarcinoma; SDR, second-degree blood relative.

Participants were followed for 63 months on average (SD 43.2). The total follow-up time was 1930 person-years. Over the span of 13 years, surveillance was ended as per protocol in 15 (4%) participants, because the stopping-age (75 years) was reached (n=13) or co-morbidities precluding pancreatic resection developed (n=2). Six individuals (2%, four mutation carriers and two FPC kindreds) died of other causes than PDAC and 36 (10%) dropped out of the programme of their own accord (reasoning specified in online supplemental eTable1).

### Protocol adherence

The 366 participants underwent a total of 1855 surveillance visits (median 4 visits per individual, IQR 6, range 1–13) and 3509 imaging tests (median seven tests per individual, IQR 12, range 1–26). There were 366 baseline visits and 1367 scheduled annual follow-up visits. In 92% of these, both EUS and MRI/MRCP were performed, in 5% only MRI/MRCP and in 3% only EUS. Reasons for deviating from the protocol included personal wish of the participant, insufficient visualisation of the pancreas on EUS in previous visits and contraindications for EUS or MRI/MRCP. In addition to these visits, there were 114 planned visits after a shortened interval of 3 or 6 months because of pancreatic abnormalities and 8 unplanned interval visits because individuals developed symptoms (EUS and MRI/MRCP in 1, EUS and CT in 2 and CT only in 5). Of the total 1481 planned follow-up visits, 81% were performed within the designated month, 9% in the month thereafter, 4% in the second month, 5% between 3 and 11 months later and 1% skipped a full year. The median interval deviation was 0.12 months (IQR 1, range 10–36).

### Pancreatic cancer incidence

**Figure 1:**
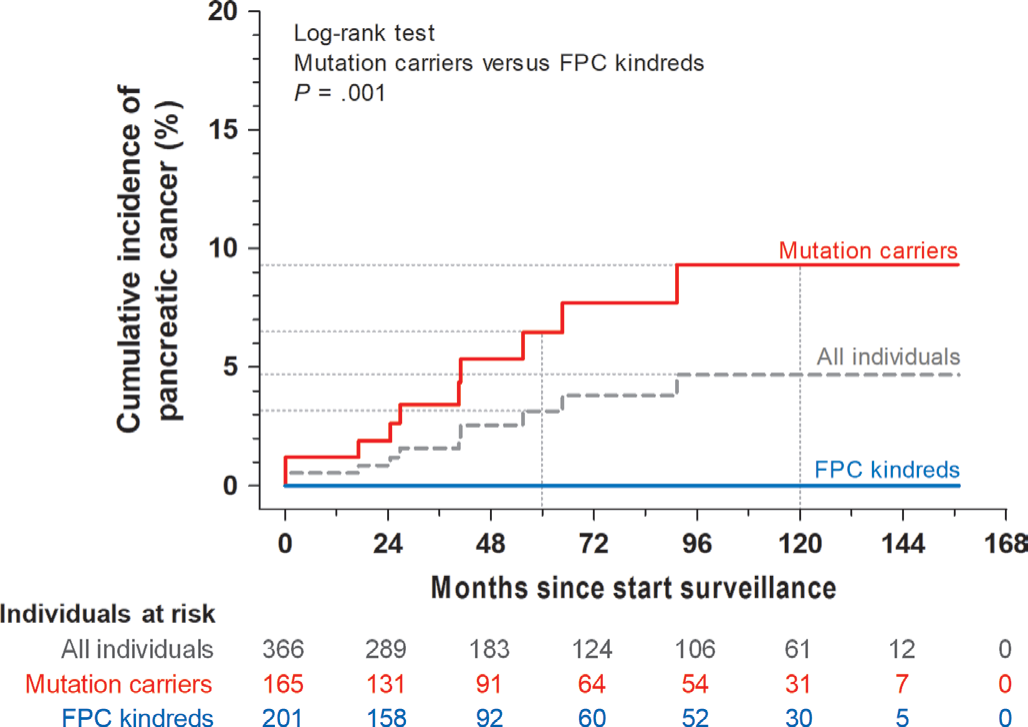
Cumulative incidence of pancreatic cancer stratified for genetic risk category. FPC, familial pancreatic cancer.

Overall, the cumulative PDAC incidence was estimated at 3.1% (8 cases) after 5 years and 4.7% (10 cases) after 10 years of follow-up (figure 1). None of the cases were FPC kindreds. Thus, the cumulative PDAC incidence in this subgroup was 0%, while in the mutation carriers group it was estimated at 6.5% at 5 years and 9.3% at 10 years (p=0.001). The characteristics of the 10 patients with PDAC are presented in tables 2 and 3, their median age at diagnosis was 61 (IQR 16.5) years. Of these, two asymptomatic patients underwent resection for a lesion diagnosed at baseline (#6 and #7 in table 2). The other eight cases developed during surveillance (incident PDACs) and were diagnosed after a median follow-up of 40 (IQR 38) months.

**Table 2:** Details of participants who developed pancreatic cancer or underwent pancreatic resection

Pathological outcome*		Age at diagnosis, years	Months between baseline and diagnosis/surgery	Symptomatic interval PDAC	Risk category	Modality that detected lesion	Modality performed but lesion not visible	FNA	Surgery type	TNM stage AJCC 8th edition	Outcome/months survival since PDAC diagnosis or surgery
*Participants with non-resectable malignancy*											
1	PDAC	65	40/–	Y	PJS	EUS, MRI/MRCP	–	N/A	–	T4N1M0	Deceased/4
2	PDAC	74	91/–	Y	*BRCA2*	MRI/MRCP†	–	N/A	–	T3N1M1	Deceased/1
3	PDAC	55	64/–	Y	*CDKN2A*	EUS, MRI/MRCP	–	N/A	–	T2N1M1	Deceased/2
4	PDAC	68	17/–	Y	*CDKN2A*	EUS, CT‡	–	N/A	–	T4N1M0	Alive/10
*Participants with resected malignancy*											
5	PDAC, R0 resection§	68	39/43	N	*CDKN2A*	EUS, MRI/MRCP, CT	–	?	DP	T1cN0M0	Deceased/12
6	PDAC, R0 resection	76	0/3	N	*CDKN2A*	EUS, CT	–	N/A	DP	T3N2M0	Deceased/32
7	PDAC, R0 resection	51	0/3	N	*CDKN2A*	EUS	MRI/MRCP, CT	N/A	DP	T1cN0M0	Deceased/32
8	PDAC associated with IPMN, R1 resection**	50	24/25	N	*CDKN2A*	EUS, MRI/MRCP¶	–	N/A	DP	T1cN1M0	Deceased/18
9	PDAC, R0 resection	54	26/29	N	PJS	EUS, MRI/MRCP¶	–	Pos	PD	T1aN0M0	Deceased/ 21††
10	PDAC, R0 resection	55	55/58	N	*CDKN2A*	EUS	MRI/MRCP, CT	Pos	PD	T2N1M0	Alive/16
*Participants who underwent resection without harbouring malignancy*											
11	Main duct IPMN, IGD	47	0/28	–	*BRCA2*	EUS, MRI/MRCP	–	N/A	DP	–	Alive/81
12	Mixed type IPMN, LGD	64	0/5	–	FPC	EUS, MRI/MRCP	–	N/A	PD	–	Alive/33
13	Branch duct IPMN, LGD+pNET G1 T1N0M0	49	50/51	–	FPC	EUS	MRI/MRCP	N/A	DP	–	Alive/44
14	Duodenal NET G2 T2N0M0 <2 cm	57	0/3	–	FPC	EUS, MRI/MRCP, CT	–	Pos	PD	–	Alive/55
15	pNET G2 T1N0M0 <2 cm	51	0/9	–	*CDKN2A*	EUS, MRI/MRCP	CT	Pos	PD	–	Alive/39
16	PanIN2+pNET G1 T1N0M0 <2 cm	49	49/50	–	*CDKN2A*	EUS	MRI/MRCP	N/A	DP	–	Alive/83
17	PanIN2	47	0/17	–	FPC	EUS, MRI/MRCP	–	N/A	DP	–	Alive/98
18	PanIN1	46	0/5	–	FPC	EUS	MRI/MRCP	N/A	PD	–	Alive/126
19	PanIN1	56	0/3	–	FPC	EUS	MRI/MRCP	N/A	PD	–	Alive/135
20	Autoimmune pancreatitis type 2	32	24/25	–	FPC	EUS, MRI/MRCP	CT	N/A	DP	–	Alive/39
21	No lesion detectable in resected specimen	50	14/16	–	*BRCA2*	EUS	MRI/MRCP	Pos	DP	–	Alive/43

*Highest grade of dysplasia reported in case of multiple lesions.

†Underwent surveillance with only MRI because the pancreas was difficult to visualise on EUS.

‡Individual had refused MRI.

§Developed pancreatic cancer outside the surveillance programme, 3 years after quitting for unknown reasons.

¶EUS detected high-risk feature that led to surgery, MRI did not.

**Pathology showed a moderately differentiated tubular adenocarcinoma, most likely originating from an IPMN.

††Died due to metastases most likely originating from cervical cancer that was diagnosed prior to the pancreatic ductal adenocarcinoma.

*AJCC*, American Joint Committee on Cancer; DP, distal pancreatectomy; EUS, endoscopic ultrasonography; FNA, fine-needle aspiration; FPC, familial pancreatic cancer kindred; IGD, intermediate-grade dysplasia; IPMN, intraductal papillary mucinous neoplasm; LGD, low-grade dysplasia; MRI/MRCP, MRI/magnetic resonance cholangiopancreatography; N, no; N/A, not applicable, FNA was not performed; NET, neuroendocrine tumour; PA, pathology; PanIN, pancreatic intraepithelial neoplasia; PD, pancreatoduodenectomy; PDAC, pancreatic ductal adenocarcinoma; PJS, Peutz-Jeghers syndrome; pNET, pancreatic neuroendocrine tumour; Pos, FNA suggestive or positive for malignancy (NET or PDAC or acinar cell carcinoma); Y, yes.

**Table 3:** Predictors for the development of pancreatic cancer in univariable analysis

Variables	PDAC (n=10)	No PDAC (n=356)	P value
Patient characteristics			
Age, median (IQR)	57 (16)	54 (13)	0.75
Mutation carrier, n (%)	10 (100)	155 (44)	<0.001
History of non-pancreatic malignancy, n (%)	5 (50)	100 (28)	0.16
History of diabetes mellitus, n (%)	1 (10)	15 (4)	0.36
Smoking, n (%)	3 (30)	45 (13)	0.37
Family characteristics			
Total number of blood relatives with PDAC, median (IQR)	2 (3)	2 (1)	0.83
Blood relative with PDAC<50 years of age, n (%)	3 (30)	120 (34)	>0.99
Pancreatic abnormalities on imaging			
Solid lesion, n (%)	7 (70)	14 (4)	<0.001
Indeterminate lesion*, n (%)	1 (10)	33 (9)	>0.99
Cystic lesion, n (%)	5 (50)	188 (53)	>0.99
Cystic lesion with solid component or mural nodule, n (%)	2 (20)	3 (1)	0.006
Cystic lesion with growth speed>5 mm/year, n (%)	3 (30)	22 (6)	0.03
Main pancreatic duct 5–9 mm (with or without focal lesion), n (%)	4 (40)	17 (5)	0.001

Patient and family characteristics were assessed at baseline, pancreatic abnormalities scored if present at any visit.

*Hypoechoic or hypointense lesions of unknown significance that could not with certainty be classified as solid or cystic at diagnosis.

PDAC, pancreatic ductal adenocarcinoma.

### Clinical course of development of incident pancreatic cancer cases

Four of the eight presented as symptomatic metastasised interval carcinomas at 1 month (#1 in table 2), 3 months (#3), 5 months (#2) and 17 months (#4, this visit was overdue) after the last surveillance visit. At this prior visit, no abnormalities were visible in one individual (#4). The other three were presumed to have an unchanged multifocal branch duct IPMN. One of them (#1) had been undergoing shortened surveillance intervals during 2 years for a possible solid component, but after two negative FNAs and no further lesion development, it was decided to return to annual surveillance. One month later she then developed epigastric pain, radiating to the back, and a CT revealed a new hypodense lesion of 2 cm. In hindsight, discrete signs of the lesion were identified on the preceding MRI/MRCP 1 month before, but were still considered not to be distinct or specific enough to cause alarm. In the second individual (#2), the PDAC had arisen seemingly independent from the unchanged IPMN. The third individual (#3), in addition to the multifocal branch duct IPMN, developed a tortuous main pancreatic duct with a stricture, yet without any visible mass despite meticulous inspection by multiple radiologists and endosonographists. An extra surveillance visit after 3 months was decided on, but before that time, she developed symptoms and was diagnosed with a, by then visible, mass in the pancreas with multiple liver metastases.

There were two surveillance-detected asymptomatic T1N0M0 cases. One of them (#5) had quit the programme of her own accord and developed PDAC while under surveillance elsewhere, outside our formal protocol. The other (#9), who also had concomitant cervical cancer, had a new solid lesion with low uptake of contrast, 2 months later, it remained unchanged on repeat EUS and FNA was suggestive of malignancy.

The last two were surveillance-detected asymptomatic metastasised cases. One (#10) had no prior visible abnormalities, while the other (#8) had a multifocal branch-duct IPMN that showed growth and developed a solid component that seemed hypovascular after contrast-enhancement.

### Pancreatic cancer resectability, survival and predictors

The PDAC resectability rate was 60% (6/10) overall and 50% (4/8) for incident cases. Three out of 10 (30%) patients with PDAC were detected at an early stage (T1N0M0 with R0 resection). For the incident cases, this concerned two out of eight (25%) patients. At the time of analysis, eight patients with PDAC (2.2%) had deceased. The two living patients (both with lymph node metastasis) underwent surgery 10 (#4) and 16 months (#10) before analysis. The median survival was 18 (IQR 28) months for all cases, 12 (IQR 15) months for the 8 incident cases, 21 (IQR 14) for the 6 resected cases and 18 (IQR 9) for the 4 resected incident cases.

In the univariable analysis (table 3), development of PDAC was associated with mutation carriers (vs FPC kindreds, p<0.001), a solid lesion on any imaging (p<0.001) and a dilated main pancreatic duct of 5–9 mm (p=0.001). A cystic lesion was only associated with PDAC if a solid component or mural nodule (enhancing and non-enhancing, p=0.006) was present, or if the growth speed exceeded 5 mm/year (p=0.03).

### Surgery

After discussion by the multidisciplinary panel, 17 individuals underwent pancreatic surgery (overall pancreatic surgery rate 4.6%) at a median age of 51 (IQR 8.8) years. Detailed characteristics including the histological outcomes are listed in table 2. The reasons for surgery were: nine solid lesions (#6, #7, #9, #10, #12, #13, #18, #19 and #20); four indeterminate lesions (#15 and #21 with FNA suggestive of malignancy, #17 with possible solid component and #16 in which the patient insisted on surgery); one cyst with solid component (#8); one cyst with main duct dilation (#11); one solid duodenal lesion with FNA positive for a neuroendocrine tumour (#14) and unknown in one (#5, surveillance elsewhere). Four (24%) individuals underwent surgery following the baseline examination (#6, #7, #19 and #14), the remainder (n=13) after a median of 26 (IQR 34) months of surveillance. Lesions were resected a median of 3.3 months (IQR 5.7) after detection and a median of 1.3 months (IQR 1.45) after the multidisciplinary panel decided on the indication for surgery. Four lesions had been followed more intensively for more than 6 months prior to being resected (table 2; #8, #11, #15 and #16), only one of which proved to be PDAC (#8, T1cN1M0).

There was no surgery-related mortality. Of the 11 individuals undergoing resections without PDAC, 7 (64%) developed pancreatic exocrine insufficiency (defined as digestive complaints responding favourably to pancreatic enzyme treatment) and 1 (9%) developed insulin-dependent diabetes mellitus, within a mean follow-up of 53 (SD 38.1) months since surgery. One individual (#12 in table 2) was successfully treated for recurrent cholangitis due to an anastomotic biliary stricture.

Besides the individuals who developed PDAC or underwent surgery, 37 other individuals developed a solid or indeterminate lesion (defined as hypoechoic or hypointense lesions of unknown significance). The work-up and outcomes of these individuals are described in the online supplemental file 1.

### Sensitivity of surveillance modalities

The sensitivities of EUS and MRI/MRCP in detecting pancreatic abnormalities are shown in table 4. EUS was more sensitive than MRI/MRCP in detecting solid lesions (100% vs 22%, p<0.001). MRI/MRCP was superior to EUS in detecting cystic lesions overall (83% vs 42%, p<0.001) but less so in the subgroup of cysts larger than 1 cm (92% vs 70%, p=0.06). EUS and MRI/MRCP had equal sensitivities to detect indeterminate lesions and dilated main pancreatic ducts.

**Table 4:** Sensitivity of surveillance modalities in detecting pancreatic abnormalities

Abnormality on imaging	Total*, N	EUS	MRI/MRCP	EUS vs MRI/MRCP P value	EUS vs EUS+MRI/MRCP P value	MRI/MRCP vs EUS+MRI/MRCP P value
Solid lesions	25	100% (22/22)	22% (4/18)	<0.001	NA	<0.001
Indeterminate lesions†	36	61% (22/36)	54% (19/35)	0.85	<0.001	<0.001
Cystic lesions	463	42% (187/446)	83% (376/455)	<0.001	<0.001	<0.001
≥10 mm	38	70% (26/37)	92% (34/37)	0.06	0.001	0.25
<10 mm	424	39% (161/409)	82% (342/418)	<0.001	<0.001	<0.001
With solid component or mural nodule	5	100% (4/4)	20% (1/5)	0.13	NA	0.13
Main pancreatic ducts 5–9 mm‡	21	62% (13/21)	60% (12/20)	>0.99	0.01	0.01
Pancreatic neuroendocrine tumours	6	100% (6/6)	33% (2/6)	0.13	NA	0.13

Sensitivity was assessed at the first detection of the abnormality. The total number of abnormalities per modality (in brackets) differ if one of the modalities had not been performed at that specific visit.

*The used reference standard was presence of the abnormality on either EUS, MRI/MRCP or CT; only for neuroendocrine tumours the diagnosis was based on cytological or histological confirmation.

†Hypoechoic or hypointense lesions of unknown significance that could not with certainty be classified as solid or cystic at diagnosis.

‡With or without the presence of a focal lesion.

EUS, endoscopic ultrasonography; MRI/MRCP, MRI/magnetic resonance cholangiopancreatography; NA, not applicable because sensitivity was 100% with and without performing MRI/MRCP; PDAC, pancreatic ductal adenocarcinoma.

For most pancreatic abnormalities, combining EUS and MRI/MRCP resulted in a higher sensitivity than either of the two alone. For solid lesions, neuroendocrine tumours and cystic lesions with solid components or mural nodules, MRI/MRCP had no added value to EUS, as the diagnostic yield of EUS was already 100%. For cystic lesions greater than 1 cm, performing EUS did not add value to MRI/MRCP alone (p=0.25).

## Discussion

We prospectively investigated the long-term yield of pancreatic cancer surveillance in a large cohort of high-risk individuals. To date, this is the only published long-term surveillance programme that included both mutation carriers and FPC kindreds, of which all families have routinely undergone genetic counselling and testing and in which both EUS and MRI/MRCP were performed at every visit. Notably, participant and protocol adherence were exceptionally high. This renders this cohort ideally suited to compare the yield of surveillance between different genetic risk categories, in particular those with a proven mutation and those without, and to assess the performance of EUS and MRI/MRCP.

The cumulative incidence of PDAC in this cohort was entirely attributable to carriers of established high-risk mutations. The incidence was highest in Peutz-Jeghers syndrome patients (2/9, 22%) and *CDKN2A* mutation carriers (7/96, 7%), confirming the findings by Vasen *et al*.^20^ The high PDAC incidence rates within these groups support the recommendation that these specific mutation carriers are favourable candidates for surveillance. Of the 45 *BRCA2* mutation carriers, none developed PDAC, despite them being a median of 4 years older than the age of PDAC onset in their families at last follow-up. Earlier studies on fewer individuals with shorter follow-up reported a somewhat higher PDAC incidence of 1 or 2 cases among 6–17 individuals.^21–28^ The largest study to date followed 41 individuals of a similar age as our cohort with a *BRCA1*, *BRCA2* or *PALB2* mutation for 5.6 years, in which one (2%) developed PDAC.^29^ Our lower PDAC incidence might be attributable to our less stringent inclusion criteria. Ten of our 45 individuals had only third-degree or higher-degree relatives affected by PDAC, whereas most other studies required at least a first-degree or second-degree affected relative, thereby selecting a population at higher risk. Although we report on one of the largest cohorts of *BRCA2* mutation carriers to date, we cannot yet draw definite conclusions about their suitability for PDAC surveillance, as is true for the other lower-risk genetic mutations.

An important observation is the fact that in over 200 mutation-negative FPC kindreds with a mean baseline age of 56 years (SD 9.8) and a mean follow-up of over 5 years, none developed PDAC. Based on this observation, the PDAC risk of mutation-negative FPC kindreds seems lower than the previously estimated risk of untested FPC kindreds.^8 10 11 30 31^ Several short-term surveillance studies have reported varying incidences in FPC kindreds of 0%–5%.^22 24–27 32–37^ However, because most studies did not account for the possible presence of genetic mutations, these incidence rates are not representative for mutation-negative FPC kindreds but apply only to untested FPC kindreds. This was recently demonstrated in a large epidemiological study.^9^ It confirmed the often-reported twofold increased PDAC risk (SIR 2.04, 95% CI 1.78 to 2.31) for first-degree relatives of incident PDAC patients, but this risk was lower in the subgroup of first-degree relatives of only mutation-negative patients (SIR 1.77, 95% CI 1.51 to 2.05), as compared with those of mutation carriers (SIR 4.32, 95% CI 3.10 to 5.86). The only long-term published surveillance study, performed by Canto and colleagues in Baltimore, USA, initially reported a PDAC incidence in untested FPC kindreds of 4.4% (13/297).^29^ However, when they retrospectively genetically tested all participants, 5 of the 13 cases actually harboured a genetic mutation, thereby decreasing the PDAC incidence in true mutation-negative FPC kindreds to 2.4% (8/330).^38^ The two other surveillance programmes incorporating routine genetic testing (in Marburg, Germany and Madrid, Spain) have not yet reported their long-term results. In Marburg, after a mean follow-up of 2.8 (range 0–10) years, they reported only one patient with PDAC in 184 (0.5%) mutation-negative FPC kindreds.^20 39^ In Madrid, after 2 years of surveillance of 24 mutation-negative FPC kindreds, none had developed PDAC.^40^ Contrary to what was previously thought, these and our data imply that mutation-negative FPC kindreds may not exceed the current threshold of 10% lifetime risk required for inclusion in our surveillance programme. Possibly, the identification of (combinations of) novel pathogenic variants associated with PDAC may provide a more precise estimation and stratification of lifetime PDAC risk in this group.^41^ Also, their PDAC risk may increase with age. Currently, there is no consensus on the age to start surveillance in FPC kindreds.^4^ The mean baseline age of the cohort from Baltimore (58 years), Marburg (48 years) and our cohort (56 years) is young compared with the mean age at which PDAC usually occurs in sporadic cases (70 years).^1^ Although at last follow-up, our FPC kindreds were a median three (IQR 16) years older than the age of PDAC onset in their families. Based on the results of these studies, regardless of whether mutation-negative FPC kindreds are eligible candidates for PDAC surveillance based on their actual lifetime risk, a starting age of 50 seems inappropriate. For the purpose of re-evaluating the yield of surveillance in FPC kindreds, a starting age of 55 or even 60 seems more fitting.

The median overall survival of PDAC (resectable and non-resectable combined) in our cohort (18 months, IQR 28, 1-year survival of 67%) compares favourably to that of sporadic PDAC outside surveillance programmes (1-year survival of 27%).^42^ Although encouraging, this difference may be (partly) explained by lead-time bias. Nevertheless, the PDAC resectability rate (60%) was also higher than that for sporadic pancreatic cancer (18%), which is associated with better outcomes.^1^ On further scrutiny, only 30% (3/10) of our patients with PDAC met the formal goal of surveillance as defined by the international CAPS consortium, namely a mass confined to the pancreas with negative margins after resection.^4^ The only two other prospective programmes in high-risk individuals with a mean follow-up greater than 3 years, by Vasen *et al* and Canto *et al*, reported similar results. Their resectability rates were 75% and 71%, respectively. Of their patients with neoplastic progression, 36% (Vasen *et al*) and 7% (Canto *et al*) could be classified as a surveillance success according the formal CAPS surveillance goals,^20 29^ for being detected and treated as high-grade precursor lesion or as PDAC confined to the pancreas with negative resection margins.

In this study, we performed more than 2700 imaging tests at more than 1400 follow-up visits and found only 8 incident PDAC cases, of which 4 presented as symptomatic metastasised interval carcinomas in between visits. These results show that although the fact that we are able to detect asymptomatic resectable PDACs, detecting the disease before it has spread beyond the pancreas is challenging, let alone to timely detect lesions with high-grade dysplasia only. Although several features were indicative of PDAC, by the time these features became apparent on imaging, the majority of individuals already had advanced disease.

It should also be noted that a minority (38%) of individuals with a solid lesion on imaging had PDAC and only a minority (35%) of individuals undergoing surgery for a suspicious lesion had malignancy or high-grade dysplasia in the resected specimen. Recently, a meta-analysis described the yield of 16 cohort studies performing surveillance in 1660 high-risk individuals, in which 257 individuals underwent pancreatic surgery but only 59 high-risk lesions were identified.^14^ These results underline the difficulty of correctly identifying relevant pancreatic lesions based on imaging, the high rates of unnecessary pancreatic surgery and the narrow window of opportunity for timely diagnosis and treatment. All this brings to question if the goals of surveillance can be reached with imaging alone and if they outweigh the harms, including the high mortality and morbidity associated with surgery. It also emphasises the importance of exploring the utility and yield of biomarkers in serum and secretin-stimulated pancreatic juice collected by duodenal sampling, in order to reduce unnecessary surgery and improve outcomes.

EUS was superior to MRI/MRCP in detecting lesions with a solid aspect and performing MRI/MRCP did not add value, while MRI/MRCP was more sensitive for sub-cm cystic lesions. This confirms the results of our previous blinded comparative analysis in a now much larger dataset.^13^ Performing MRI/MRCP in addition to EUS resulted in a higher diagnostic yield of indeterminate lesions, cystic lesions and dilated main pancreatic ducts. However, the clinical relevance of these observations is unclear. There are insufficient data regarding the progression rate and potential development of these abnormalities into clinically relevant lesions, that is, high-grade dysplastic lesions and (early) cancer. In two patients with T1 PDAC (#8 and #9 in table 2), both modalities identified the cystic lesion, but only EUS detected the high-risk stigmata that led to surgery. Two additional early-stage PDAC cases in our cohort (#7 and #10), and two described by Canto *et al*, were detected by EUS while not visible on MRI/MRCP and CT.^29^ Although numbers are small, these results suggest that EUS is superior to MRI/MRCP in detecting malignancies at the earliest possible stage. Together with the opportunity to collect secretin-stimulated pancreatic juice from the duodenum for research purposes, this has led our team to start implementing EUS as our standard surveillance modality and to perform MRI/MRCP only at baseline and on indication during follow-up.

Though being the largest study to date, this study is still limited by the low number of cases. Also, a longer follow-up would yield more cases, enabling a more reliable risk assessment in the various subgroups and a better assessment of the harms and benefits of surveillance. It would also allow drawing more definite conclusions regarding the yield of surveillance in mutation-negative FPC kindreds. It should be acknowledged that the mutation-negative FPC kindreds currently under surveillance are relatively young and that their risk to develop PDAC will increase with older age. In addition, in this study, genetic testing was performed on a PDAC index case in 99 of the 201 FPC kindreds. In the 70 families of the other 102 individuals, genetic mutations may have been missed. This could have led to unjust inclusion of individuals who did not inherit the familial risk, thereby lowering the PDAC risk in the FPC kindreds group. However, the chance of identifying a genetic mutation in a PDAC index case is under 20%, even in FPC families.^43–45^ Even if a genetic mutation was missed in 20% of these families, our cohort would still include more true mutation-negative FPC kindreds than mutation carriers, thereby leaving our conclusions on the comparison of PDAC risk and eligibility for pancreatic cancer surveillance intact. A limitation of all PDAC surveillance studies is the lack of a control arm, preferable with individuals from the same families, who do not undergo surveillance. Although a randomised controlled trial would methodically be best suited to assess a potential survival benefit, ethical considerations prohibit such a study design.

In conclusion, the diagnostic yield of PDAC was substantial in high-risk mutation carriers, in particular in *CDKN2A* mutation carriers and patients with Peutz-Jeghers syndrome, but non-existent in the mutation-negative FPC kindreds. Whether there is a benefit of surveillance for FPC kindreds at an older age remains to be proven. Timely identification of resectable lesions proved challenging despite the concurrent use of two imaging modalities, with EUS outperforming MRI/MRCP. Although some indicators suggest a diagnosis at an earlier stage and a longer median survival with surveillance, lead-time bias cannot be excluded and a genuine survival benefit cannot be ascertained. Overall, surveillance of individuals at high risk of developing PDAC by imaging yields suboptimal results and in order to improve outcomes, it is pivotal to explore other diagnostic modalities including biomarkers.

## Data Availability

All data relevant to the study are included in the article or uploaded as supplementary information.
