# Supplementary material for: Long-term yield of pancreatic cancer surveillance in high-risk individuals
Source: Gut. 2021 Apr 5;71(6):1152–60. doi: 10.1136/gutjnl-2020-323611 (PMC9120399; doi:10.1136/gutjnl-2020-323611)
Supplement: Supplementary data [file gutjnl-2020-323611supp001.pdf]

Online-Only Supplements

eMethods

Inclusion criteria Page 2.
Exclusion criteria Page 2.
Age criteria Page 2.

eResults

Participant drop-out Page 3.
Solid and indeterminate lesions Page 3.

Overbeek KA, *et al. Gut* 2022; 71:1152–1160. doi: 10.1136/gutjnl-2020-323611

eMethods

Inclusion criteria

Participants had to meet one of the following inclusion criteria:

1) Carry a *CDKN2A* gene mutation affecting the p16INK4A protein, regardless of pancreatic ductal adenocarcinoma (PDAC) family history
2) Have Peutz-Jeghers syndrome (proven *LKB1/STK11* gene mutation or clinical diagnosis), regardless of PDAC family history
3) Carry a *BRCA2*, *BRCA1*, *TP53*, *MLH1*, *MSH2* or *MSH6* gene mutation, and have ≥ 2 blood relatives with PDAC, of which ≥ 1 histologically proven
4) Be a first-degree blood relative of a family member with PDAC, in a family with ≥ 1 histologically proven PDAC, and either:

   a) PDAC in ≥ 2 blood relatives who were first-degree relatives to each other
   b) PDAC in ≥ 3 blood relatives, who were first or second-degree relatives to each other
   c) PDAC in ≥ 2 blood relatives, of whom ≥ 1 was under 50 years of age, who were first or second-degree relatives to each other

Exclusion criteria

Participants were excluded if they either:

1) Had a personal history of PDAC
2) Were under 18 years old
3) Were unable to provide informed consent due to mental retardation or a language barrier
4) Had an upper gastrointestinal tract obstruction or stricture not allowing passage of the echoendoscope
5) Had an American Society of Anesthesiologists score ≥ 3

Age criteria

The minimum age of inclusion was 45 years until 2013 and 50 thereafter, or ten years younger than the age of the youngest relative diagnosed with PDAC, whichever was lowest. For individuals with Peutz-Jeghers syndrome (*LKB1/STK11*) the minimum age of inclusion was 30 or ten years younger than the youngest PDAC onset-age in the family. Surveillance ended at the age of 75.

eResults

eTABLE 1. Participant drop-out

**Participation status, n (%)**	**Participants (N=366)**
Current participant in study	309 (8.4)
Surveillance ended per protocol	15 (4.1)
Reached stopping-age	13 (3.5)
Developed co-morbidities	2 (0.5)
Died of other cause than PDAC	6 (1.6)
Dropped out	36 (9.8)
Lost to follow-up	6 (1.6)
Quit without giving reason	10 (2.7)
Quit with giving one or more reasons	20 (5.5)
Physical burden	2 (0.5)
Psychological burden	5 (1.4)
Costs	7 (1.9)
Logistic reasons (like travel distance)	5 (1.4)
Had enough of surveillance	3 (0.8)
Unhappy with level of care	1 (0.3)

Solid and indeterminate lesions

During the complete follow-up period, imaging detected 25 solid lesions in 21 individuals (6%) and 36 indeterminate lesions in 34 individuals (9%, defined as hypoechoic or hypointense lesions of unknown relevance that could not with certainty be classified as cystic or solid at diagnosis). The combined incidence was not different between FPC kindreds and mutation carriers (14% versus 15%, *P*=·85).

For the evaluation of these lesions, the 54 individuals underwent zero to eight additional visits (median 1, IQR 2) and zero to nine additional investigations (median 1, IQR 3). Eight individuals (15%) were diagnosed with PDAC (Table 2; #1, #2, #3, #4, #6, #7, #9 and #10, described earlier). All eight had a solid lesion (38% of the individuals with a solid lesion), while none of the individuals with an indeterminate lesion had PDAC.

Nine other individuals (17%) underwent surgery but did not have PDAC or high-grade precursor lesions (Table 2; #12, #13, #15, #16, #17, #18, #19, #20 and #21), five of which had a solid lesion and four an initially indeterminate lesion. In the remaining 37 individuals, the lesion could either no longer be detected on follow-up investigations (16), was determined to be a neuroendocrine tumor (3), ectopic spleen tissue (1), a non-pathological lymph node just outside the pancreas (1), a cystic lesion (3), physiological parenchyma (3) or pancreatic steatosis (7), or remained indeterminate but was stable in size and not suspect for malignancy (3). These 37 individuals returned to regular annual surveillance and were followed a median 31 months (IQR 57, range 0-144) since diagnosis of the lesion, in which time none presented with symptomatic PDAC.

Overbeek KA, *et al. Gut* 2022; 71:1152–1160. doi: 10.1136/gutjnl-2020-323611
